# Up Scalable Full Colour Plasmonic Pixels with Controllable Hue, Brightness and Saturation

**DOI:** 10.1038/s41598-017-01266-6

**Published:** 2017-04-26

**Authors:** Renilkumar Mudachathi, Takuo Tanaka

**Affiliations:** 1 Metamaterials Laboratory, RIKEN, 2-1 Hirosawa, Wako, Saitama 351-0198 Japan; 2 Innovative Photon Manipulation Research Team, RIKEN Center for Advanced Photonics, 2-1 Hirosawa, Wako, Saitama 351-0198 Japan; 3 School of Materials and Chemical Technology, Tokyo Institute of Technology, Tokyo, 152-8550 Japan

## Abstract

It has long been the interests of scientists to develop ink free colour printing technique using nano structured materials inspired by brilliant colours found in many creatures like butterflies and peacocks. Recently isolated metal nano structures exhibiting preferential light absorption and scattering have been explored as a promising candidate for this emerging field. Applying such structures in practical use, however, demands the production of individual colours with distinct reflective peaks, tunable across the visible wavelength region combined with controllable colour attributes and economically feasible fabrication. Herein, we present a simple yet efficient colour printing approach employing sub-micrometer scale plasmonic pixels of single constituent metal structure which supports near unity broadband light absorption at two distinct wavelengths, facilitating the creation of saturated colours. The dependence of these resonances on two different parameters of the same pixel enables controllable colour attributes such as hue, brightness and saturation across the visible spectrum. The linear dependence of colour attributes on the pixel parameters eases the automation; which combined with the use of inexpensive and stable aluminum as functional material will make this colour design strategy relevant for use in various commercial applications like printing micro images for security purposes, consumer product colouration and functionalized decoration to name a few.

## Introduction

Recently plasmonic nano structures have been actively explored for structural colour production, which was earlier, demonstrated by photonic crystals, inspired by Mother Nature^1–5^. Printing colour using plasmonic nano structures has several advantages over the conventional pigment based colouration in terms of printing resolution, robustness and reliability, compactness, compatibility of integration and functionalization, chemical hazardousness and resource requirements, despite being limited to micro scale colour image printing. The plasmonic approach stand one step closer to the real pigment based colouration in principle, owing to their preferential photon absorption and scattering arising from the resonant interaction with electromagnetic waves, which excites surface plasmon modes along the metal dielectric interface, a collective oscillation of free electron gas^6, 7^. A seminal work on reflection mode plasmonic colour production was reported by Kumar *et al*. with an emphasis on high resolution colour printing applications^8^, followed by several others focused on the optimization of plasmonic colour pixels for advanced colour printing technologies^9–11^, reflection mode colour filtering^12^, consumer product colouration, tunable structural colours, and vivid full colour generation for possible applications like anti-counterfeiting devices, security labels, information storage, stenography and functionalized decoration^13–15^. The generation of all colours in the visible spectrum with distinct reflective peaks is highly desirable for any kind of colour printing applications. So far the colours produced in most of the aforementioned works are complementary colours of the broad reflection spectra. However, colours with distinct reflective peaks have been reported by using complex geometries^12, 16–18^, which are difficult to mass-produce using roll – to – roll nanoimprinting technique^19^.

Polarization and viewing angle insensitivity, tunability across the operating wavelength range, scalable fabrication and mechanical and colour durability are among the mostly addressed issues related to this approach^8–15^. The production of full range of colours spanning the entire visible region with controllable colour attributes such as hue, brightness and saturation is also one of the most important requirements for this field to become useful in many practical applications. Therefore the challenge here is to design a simple plasmonic colour printing approach that addresses these issues and simultaneously guarantees low cost and high throughput scalable fabrication. Sub 100 nm metal structures are employed as plasmonic pixels in many of the reported works^8–17, 20^. The smaller structures demand tight control over several parameters during fabrication involving electron beam lithography (EBL) and nanoimprint lithography (NIL), which is a bottleneck for large scale implementation, even though they ensure near diffraction limit printing resolution. Such a high printing resolution is not really desired for many practical applications like consumer product colouration, functionalized decoration and macro scale colour image printing. Instead, relatively larger structures considerably relax the fabrication requirements. Here we present an up scalable colour printing scheme using plasmonic pixels of single constituent metal structure of sub-micrometer size, enabling the design of full colours with controllable colour attributes suitable for aforementioned applications.

**Figure 1:**
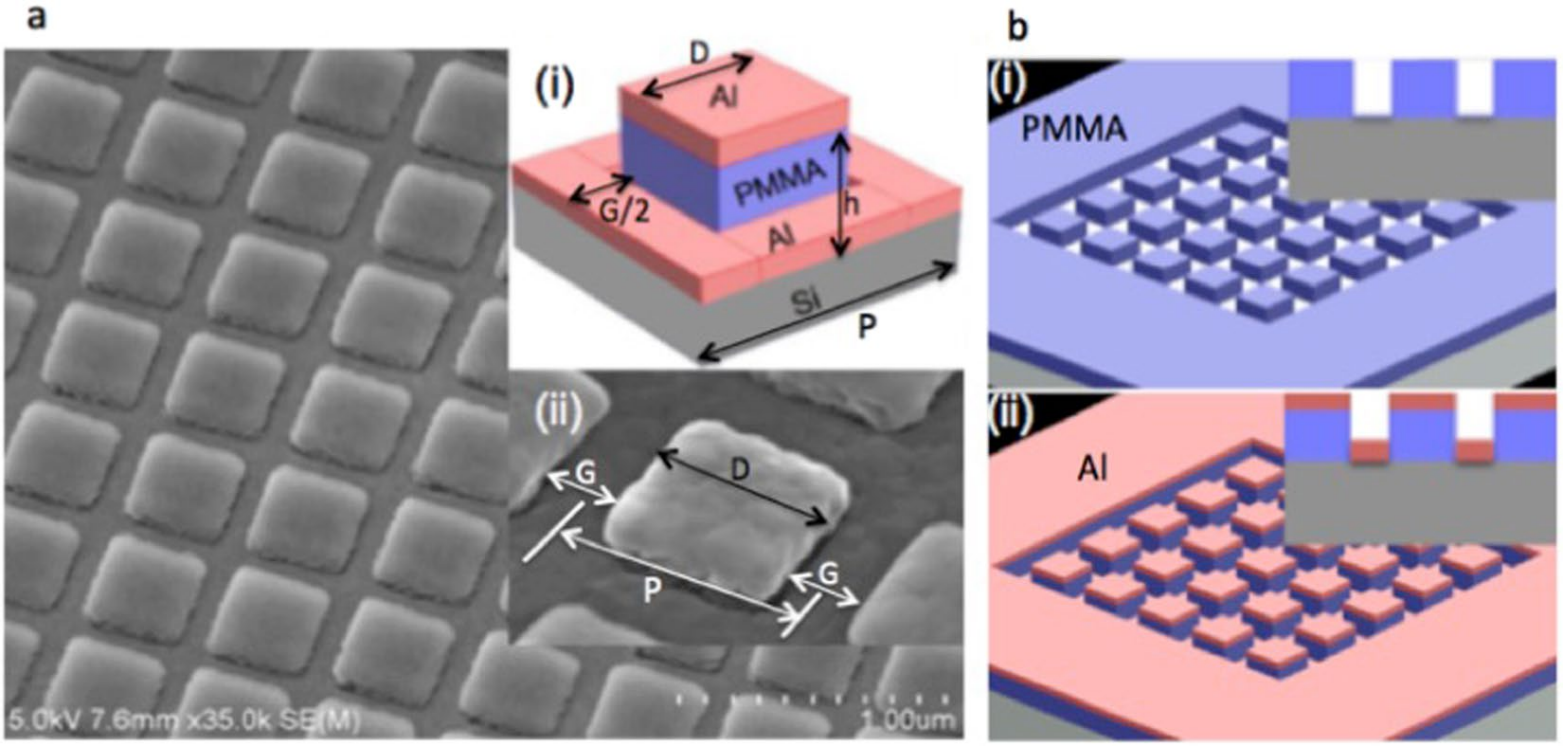
SEM image of the plasmonic pixels in the 2D array structure and its simple fabrication process steps. (**a**) SEM image of the Al square patches hovering on PMMA nano pillars periodically arranged in a square lattice in the background of a fishnet like Al back reflector, insets (i) shows the schematic rendering of a single plasmonic color element and (ii) the slightly tilted (tilt angle of 20°) close-up SEM image of the single colour element. (**b**) Simple fabrication process involving (i) the patterning of 150 nm thick PMMA resist spin coated onto a piece of (100) silicon wafer using EBL. The exposed area of the resist is removed in a developer solution leaving PMMA nano pillars (inset shows the cross-sectional view). (ii) A 45 nm thick aluminum thin film is thermally evaporated onto the nano pillars for realizing vivid full colour pallets.

We started with analyzing plasmonic colour elements comprising metal square structures of size ranging from 260 nm, which is greater than or almost equivalent to the optical diffraction limit if 500 nm is assumed as the mid spectrum wavelength. A simple geometry that requires a single layer fabrication process employing EBL and metal deposition is chosen for this study. As shown in the inset of Fig. 1a the single plasmonic colour pixel is made of a metal square patch raised on top of polymer nano pillar in the background of a perforated metal back reflector. In the single layer fabrication process, the nano pillars were defined in a 150 nm thick positive tone polymethyl methacrylate (PMMA) resist using EBL followed by the deposition of 45 nm thick aluminum thin film by thermal evaporation, which is schematically represented in the Fig. 1b and detailed in the method. We employed aluminum as the functional material for the realization of full colour pallets mainly due to its relatively high plasma frequency that facilitates colour rendering in the violet-blue region, which is never possible with gold. Also low cost and abundance of aluminum makes it the widely used material in the industrial processes. Besides, aluminum facilitates the lossy interband transition outside visible spectrum, which is crucial for plasmonic colour production tunable across the visible region. Apart from that, when compared with gold, aluminum does not require an adhesion layer and with silver, it does not require a capping layer, which considerably eases the fabrication processes^17, 20–24^.

## Results and Discussion

**Figure 2:**
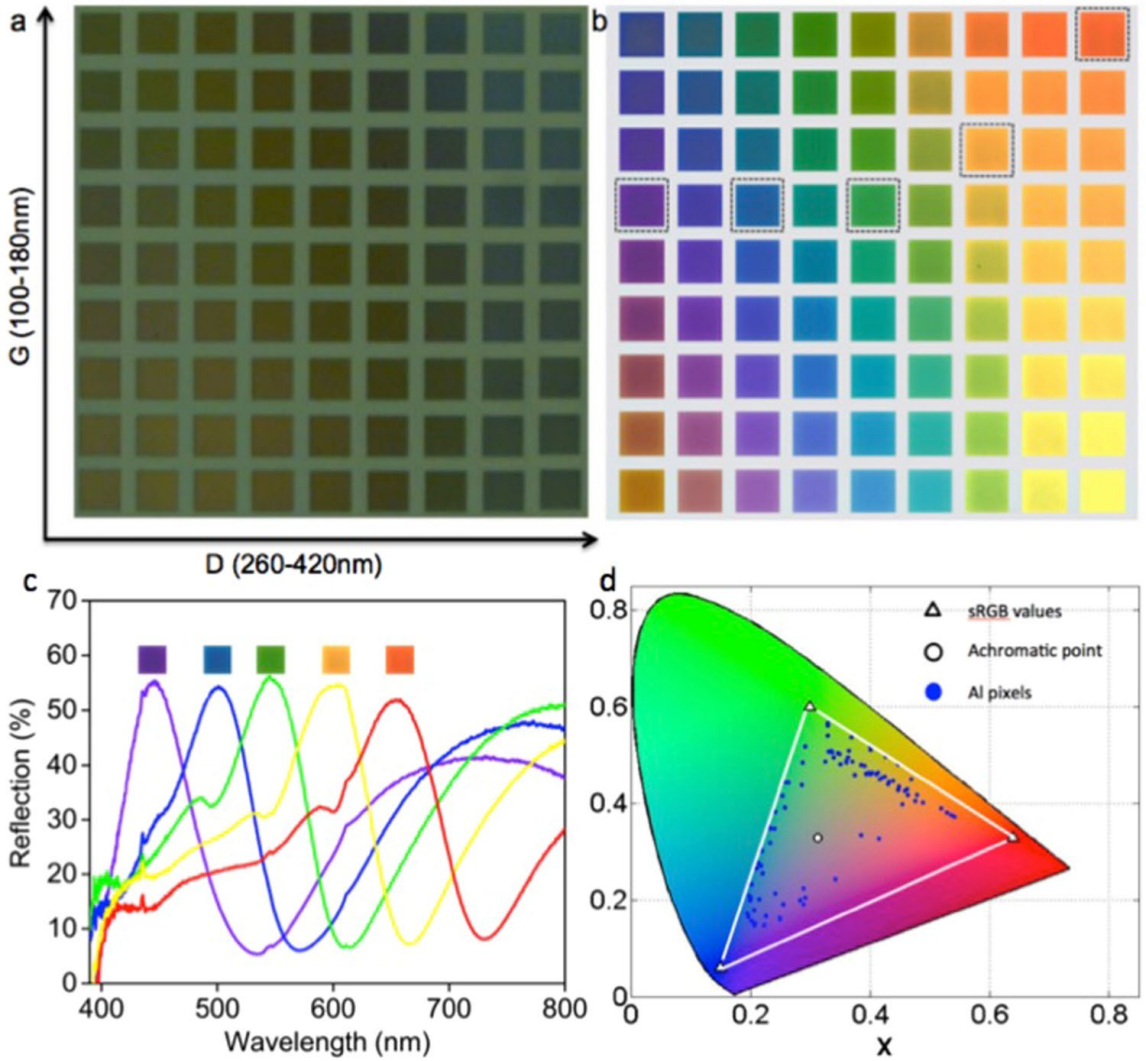
Optical micro-images and spectral analyses of the colour pallets with varying square size D and gap size G. Each pallet of 20 μm square consists of 2D arrays of sub-micron structures arranged in a square lattice. The size of the Al square patch D varies from 260 nm to 420 nm with an increment of 20 nm in the horizontal axis and the gap size G varies from 100 nm to 180 nm with an increment of 10 nm in the vertical axis. (**a**) PMMA nano pillars arranged in the square lattice show no colouration before metal deposition. (**b**) After the deposition of thin Al layer of uniform thickness, the pallets displayed bright and distinct colours depending on the geometrical parameters D, G and P. (**c**) Experimental reflection spectra of selected pallets (highlighted within the black boxes in (**b**)) captured using micro-spectroscopic technique. (**d**) Corresponding positions of all colours in (**b**) plotted in the CIE1931 colour space. The blue circles represent the tristimulus values of the aluminum colour pixels, which are scattered far from the achromatic point represented by the white circle. The blue circles formed a ring around the achromatic point confirming the capability of the proposed plasmonic geometry for the generation of full colours with high colour saturation. The x and y values for the primary colours red, green and blue are 0.5421; 0.3738, 0.3290; 0.5679 and 0.1951; 0.1722 respectively and are comparable with that of the sRGB values 0.6400; 0.3300, 0.3000; 0.6000 and 0.1500; 0.0600 common to all displays, represented by the white triangles in the figure.

In order to study the spectral behavior of the plasmonic geometry we fabricated metal square structures periodically arranged in a square lattice as shown in the Fig. 1a. Full pallets of colours are obtained by systematically varying square size D, inter particle spacing, say gap size G and pixel size P = D + G. The optical micro-images of the sample taken before and after the deposition of aluminum thin film of uniform thickness are shown in Fig. 2a,b, respectively. The pallets display full colours from violet to red with high colour saturation and brightness spanning the entire visible region.

The minimum size of a single pixel which ensures saturated colour is 440 nm assuming D = 260 nm and G = 180 nm (pure blue colour at the top left corner in Fig. 2b) and the maximum size is 600 nm assuming D = 420 nm and G = 180 nm (pure red colour at the top right corner in Fig. 2b). That is, the size of a single pixel size is comparable to the operating wavelength suitable for high-speed large-scale fabrication. It is well known that pure colour is resulted from a narrow reflective peak with its sidebands suppressed beyond detectable level in the reflection spectrum. And in the case of a printed colour image, the image quality is determined by how efficiently the individual colour elements selectively cutoff a broad range of wavelengths while allowing a narrow band to be reflected back. Therefore in the case of plasmonic colour elements broadband light absorption at multiple wavelengths are required to achieve distinct reflective peaks of high quality factor. Plasmon hybridization between the top and bottom nano structures separated by a small gap of 10–30 nm has been explored for exciting light absorption at multiple wavelengths^13, 21, 24^. In such cases, both the resonance dips must be tunable at the same scale across the spectrum to design pure colours with narrow reflection peaks. But in most of the cases, the colours displayed are light and complementary in nature mainly due to the differential tuning of the two resonance dips leading to the spectral broadening of the reflective peaks.

In contrast to the smaller gap, we explored larger pillar height for shaping the spectral features of the plasmonic colour elements. The combined plasmonic geometry in the 2D array structure is then numerically studied by systematically increasing the pillar height and found that the emergence of higher order resonance in addition to the default localized surface plasmon resonance (LSPR) of the metal square patch within the same operating wavelength range of 400–800 nm. The computer model is based on the finite difference time domain (FDTD) algorithm provided by Lumerical’s FDTD solutions as detailed in the Supplementary Information S1. It is also found from the simulations that the light extinction efficiency at these two resonance positions reaches to a maximum value for an optimum pillar height of 200 nm (see Supplementary Figs S1 and S2 for more details). The pillar height of the fabricated structures was measured to be 150 nm using atomic force microscope, see Supplementary Fig. S3 for more details and all calculations are carried out using this pillar height. Therefore, engineering the plasmonic resonances produces brilliant saturated colours with distinct reflective peaks by encoding near unity light absorption at two separate wavelengths in a single plasmonic pixel that are tunable across the visible region.

The reflection spectra measured for selected (marked in black outline) colour pallets in Fig. 2b using micro-spectroscopic technique are shown in Fig. 2c (see Supplementary Fig. S4 for the experimental spectra of other colours). The corresponding colours are also shown in the inset. The two resonance dips are more pronounced in the reflection spectra and less than 5% reflection for lower order mode and less than 35% for higher order mode are achieved, which is sufficient for suppressing the sideband reflection beyond detectable level around the peak reflection of more than 50%. The RGB values of the colours displayed in Fig. 2b were extracted using a standard algorithm from the colour images captured by the CCD camera. The chromaticity values for each colour are then calculated using these values assuming day light illumination^25, 26^. The x-y positions of each colour are then plotted in the CIE1931 chromaticity diagram as shown in the Fig. 2d. The blue circles representing the x-y positions of the colours displayed by the aluminum pixels are scattered far from the neutral point (represented by white circles) indicative of saturated colours including deep blue, green and red hues, and it forms a ring around the neutral point suggesting the capability for full colour production using this approach (see Supplementary Fig. S5 for more details). In the Fig. 2d, the white triangles represent the red, green and blue tristimulus values usual to all standard red, green and blue (sRGB) displays and the area enclosed by the white lines represents the sRGB colour gamut.

It is important to identify the physical origin of these resonances to design pure colours with similar spectral features across the operating wavelength region. It is understood from the simulations that the pillar height determines only the strength of the resonance at higher order mode and the resonance wavelength is determined by the pixel size P, which is equal to the periodicity. Similarly the light absorption at lower order mode is originated from the strong LSPR supported by the aluminum square patch and the resonance wavelength is determined by the square size D. Therefore, the colours displayed in the Fig. 2b are optimized by varying square size in the lateral direction and gap size in the vertical direction, and in both cases, the pixel size is also changed in proportion, i.e. during square size variation the gap size has been kept constant by changing the pixel size at the same scale and during gap size variation the square size has been kept constant effectively changing the pixel size.

**Figure 3:**
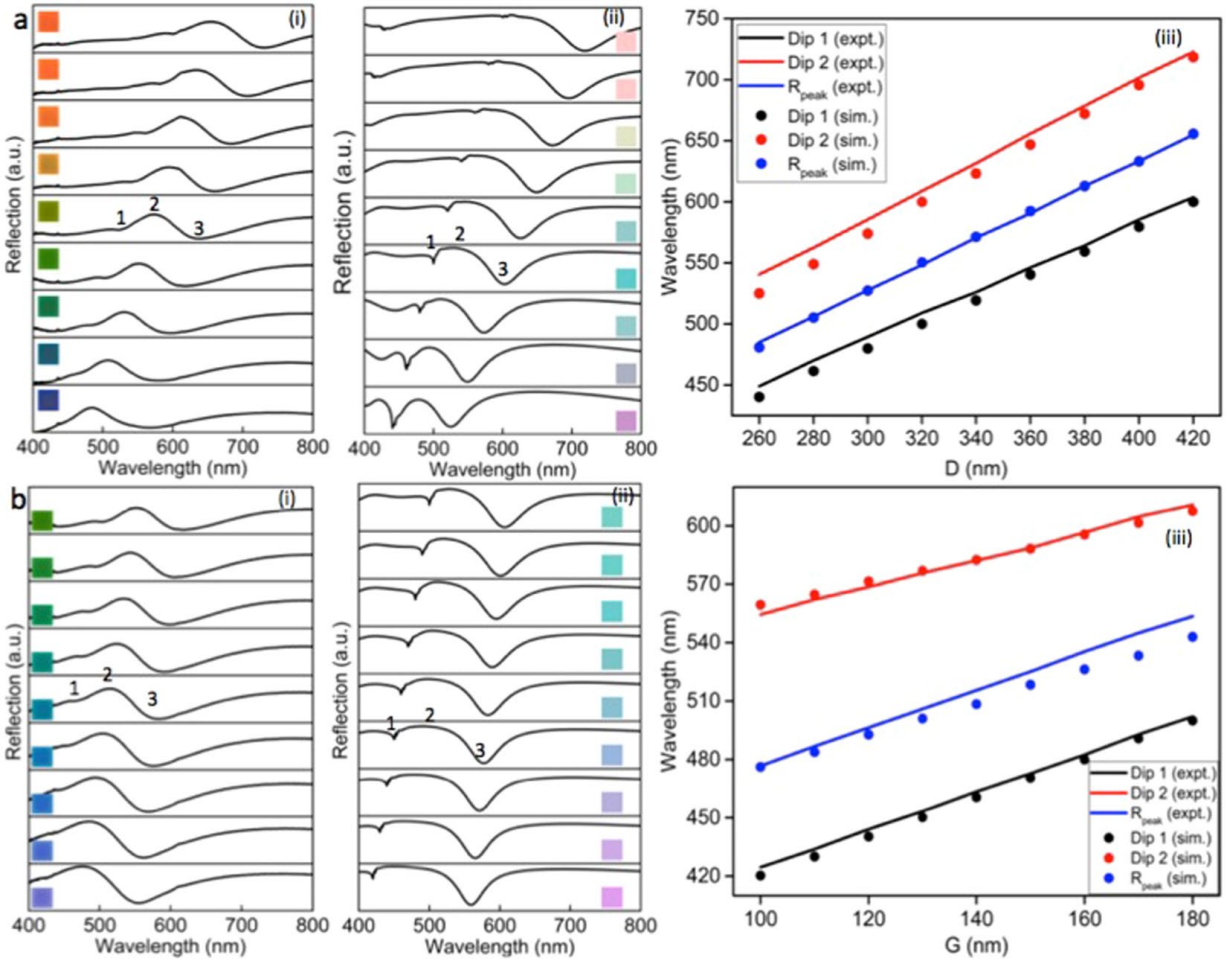
Spectral analyses of the colour pallets using micro-spectroscopic technique and a comparison between measured and simulated spectra. (**a**) Spectral analyses of selected colour pallets with varying D and fixed G, (i) experimental spectra of the colour pallets (inset shows the corresponding colours) with a fixed gap size G of 180 nm and a varying square size D from 260 nm to 420 nm, (ii) corresponding simulated spectra using FDTD algorithm with the inset shows predicted colours from the simulated spectra for each colour pallet and (iii) experimentally (lines) observed resonance dip and peak shifts as a function of D and its comparison with the simulated (dots) values. (**b**) Spectral analyses of selected colour pallets with varying G and fixed D, (i) experimental spectra of the colour pallets (inset shows the corresponding colours) with a fixed square size D of 320 nm and a varying gap size G from 100 nm to 180 nm, (ii) corresponding simulated spectra using FDTD algorithm with the inset shows predicted colours from the simulated spectra for each colour pallet and (iii) experimentally (lines) observed resonance dip and peak shifts as a function of G and its comparison with the simulated (dots) values. Positions marked as 1, 2 and 3 in all figures represent Dip1, R_peak_, and Dip2, respectively.

Two sets of analyses are presented in Fig. 3 in which we systematically study the spectral behavior of the plasmonic pixels in the 2D array structures in terms of varying D and G. Since P changes at the same scale in both the cases, here after we focus only on D and G. These analyses are aimed to trace different parameters influencing the resonances and could be manipulated to engineer important spectral attributes of a colour pixel such as hue, brightness and saturation. The measured and calculated reflection spectra of the colour pallets in the top raw of Fig. 2b, which displays saturated colours from blue to red, are plotted in Fig. 3a(i) and (ii) respectively. The inset of Fig. 3a(i) shows the respective imaged colours of the aluminum pixels and that of Fig. 3a(ii) shows the predicted colours from the calculated spectra for the respective pixels. In this case D and P are varied from 260 nm to 420 nm and 440 nm to 600 nm respectively, keeping G constant at 180 nm. As stated earlier the colour formation is based on the coexistence of broadband plasmonic light absorption at two separate wavelengths. The positions of resonance dips Dip1 and Dip2 and that of the reflection peak R_peak_ are represented as 1, 3 and 2 respectively in Fig. 3a,b. As noted the spectral features predicted by the numerical simulation are in good agreement with the experimentally observed spectra. Although the small differences may be attributed to the structural deformation due to fabrication imperfection and oblique incidence from the high numerical aperture objective lens used for the optical measurements; apart from the effect of diffractive coupling of plasmon resonance. The emergence of additional resonance dips in the 400–500 nm wavelength range for an incident angle above 10° was observed in the numerical simulations which can be attributed to the diffractive coupling of plasmon resonance and the readers are referred to the Supplementary Information S6 for more details.

In Fig. 3a(iii), the wavelength shift of the two plasmonic resonance dips Dip1 and Dip2 and that of the reflection peak R_peak_ is plotted as a function of D. It is clear that the wavelength shifts for Dip1 and Dip2 are consistent with each other attesting its dependence on the two varying parameters D and P. It is evident from the Fig. 3a(i) and (ii) that equally varying D and P guarantees narrow line width for all colour pallets in the raw with constant G. However the lines correspond to Dip1 and Dip2 are slightly diverging, indicative of spectral broadening for larger values of D and hence P, which deteriorates the colour saturation. A decreasing light absorption efficiency seen at Dip1 with increasing D is also contributed to the observed spectral broadening. In Fig. 3b, the spectral behavior of the pallets in the fourth column of Fig. 2b with a constant D of 320 nm is analyzed as a function of G. As evident from Fig. 3b(iii), the Dip1 shift is more than that of Dip2 for a given change of G, indicating its strong dependence on varying G and hence P, underlines its origin from the pixel size in the array structure. It is also noted that the lines correspond to Dip1 and Dip2 are converging suggesting a spectral narrowing for larger values of G, which provides an additional control parameter for tuning the color saturation of the pallets as seen in Fig. 3b(i) and (ii) in which the inset shows imaged and predicted colours of the respective aluminum colour pallets. Therefore hue, one of the three color properties could be tuned across the visible spectrum by varying D and P. It is also understood from these analyses that, the spectral shape of the individual colours could be preserved across the operating wavelength range by tuning D and P simultaneously at the same rate.

**Figure 4:**
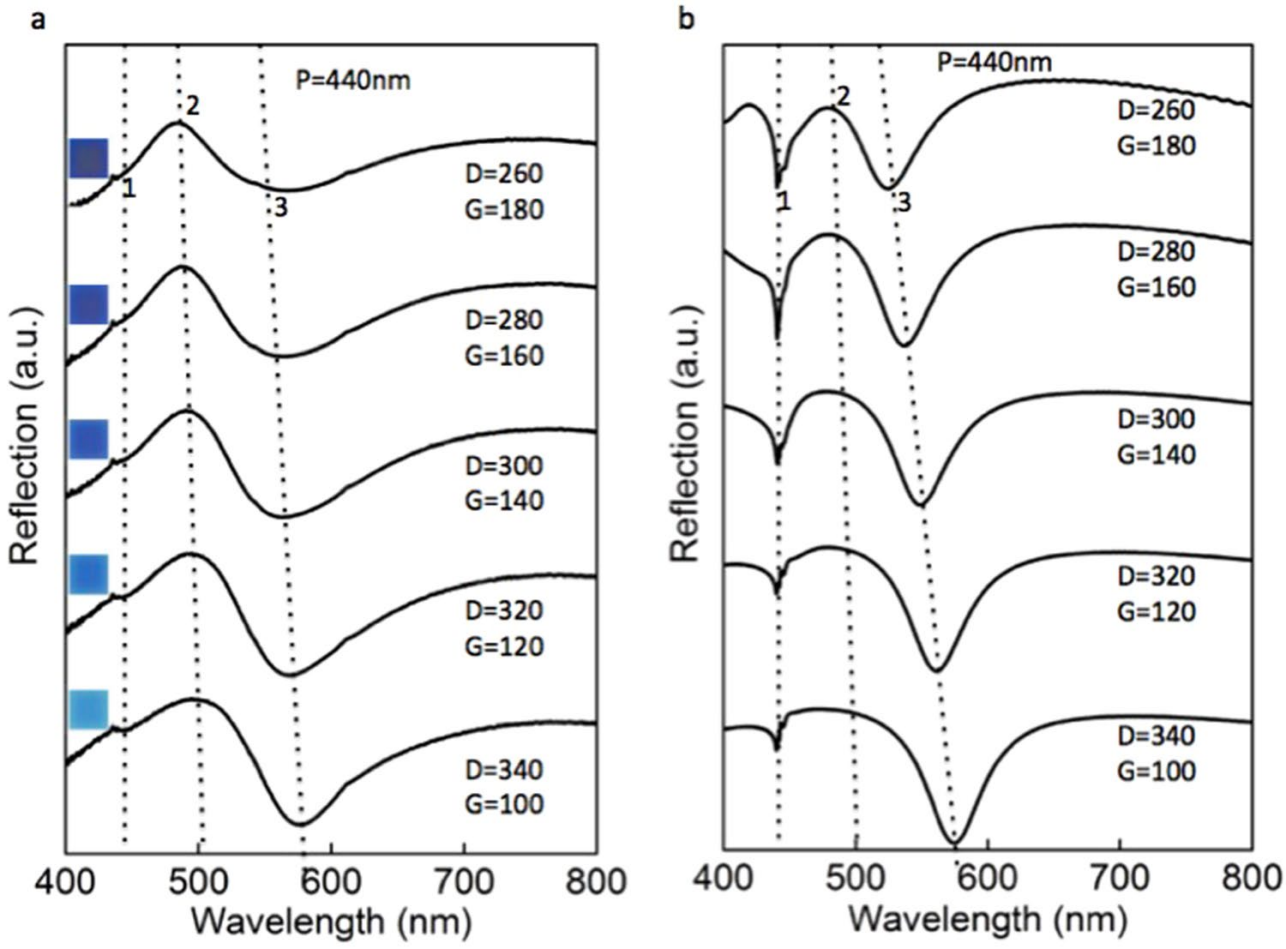
Tuning the colour saturation. (**a**) Experimental reflectance of the structures of same P with varying G and D. The gap size G was varied from 100 nm to 180 nm with an increment of 20 nm within the same pixel size P of 440 nm. Increasing G effectively reduces the square size D keeping P constant. As indicated by the dashed lines the resonance Dip2 at position 3 shifts towards the resonance Dip1 at position 1, which is invariant for changing G and D within the same P. This blue shift of the resonance Dip2 resulted in the spectral narrowing of the reflection peak R_peak_. The respective colours of the pallets shown in the inset of Fig. 4a confirmed the increasing colour saturation with increasing G, which in turn provides a means for tuning the colour saturation. (**b**) The corresponding simulated spectra of the structures show good agreement with the measured spectra.

Now we will discuss the behavior of different spectral features with respect to varying D and G for colour pallets with same pixel size P. We consider pixels of size 440 nm for this study and is presented in Fig. 4 in which the positions 1, 2 and 3 marked in both the measured and calculated spectra represent the resonance positions Dip1, R_peak_, and Dip2, respectively (see Supplementary Fig. S7 for the details of the pixels P = 480 nm). It is to be noted that the resonance wavelength 440 nm at Dip1 is invariant for changing D and G within the same P, which has the same value as that of the resonance wavelength. But as shown earlier in Fig. 3a,b, the resonance wavelength at Dip1 red shifts at the same scale with increasing P, attesting that the resonance at Dip1 is solely determined by the pixel size and hence its physical origin. The dashed lines in both the Fig. 4a (measured) and b (calculated) represent the trends of the wavelength shift at resonance positions Dip1, R_peak_, and Dip2. Increasing the gap size within constant pixel size effectively decreases the square size, which results in the blue shift of the resonance position at Dip2 as seen in both the measured and calculated spectra. This displacement of the resonance position Dip2 towards the invariant resonance at Dip1 for increasing G effectively narrow the reflection peak R_peak_. This increases the colour saturation of the pallets as indicated by the respective colours in the inset of Fig. 4a. Apart from this, decreasing peak reflection intensity with increasing G is also observed (see Supplementary Fig. S8 for more details). Therefore the gap size G provides an additional control parameter to tune the colour saturation and brightness of the pallets without changing the hue considerably.

**Figure 5:**
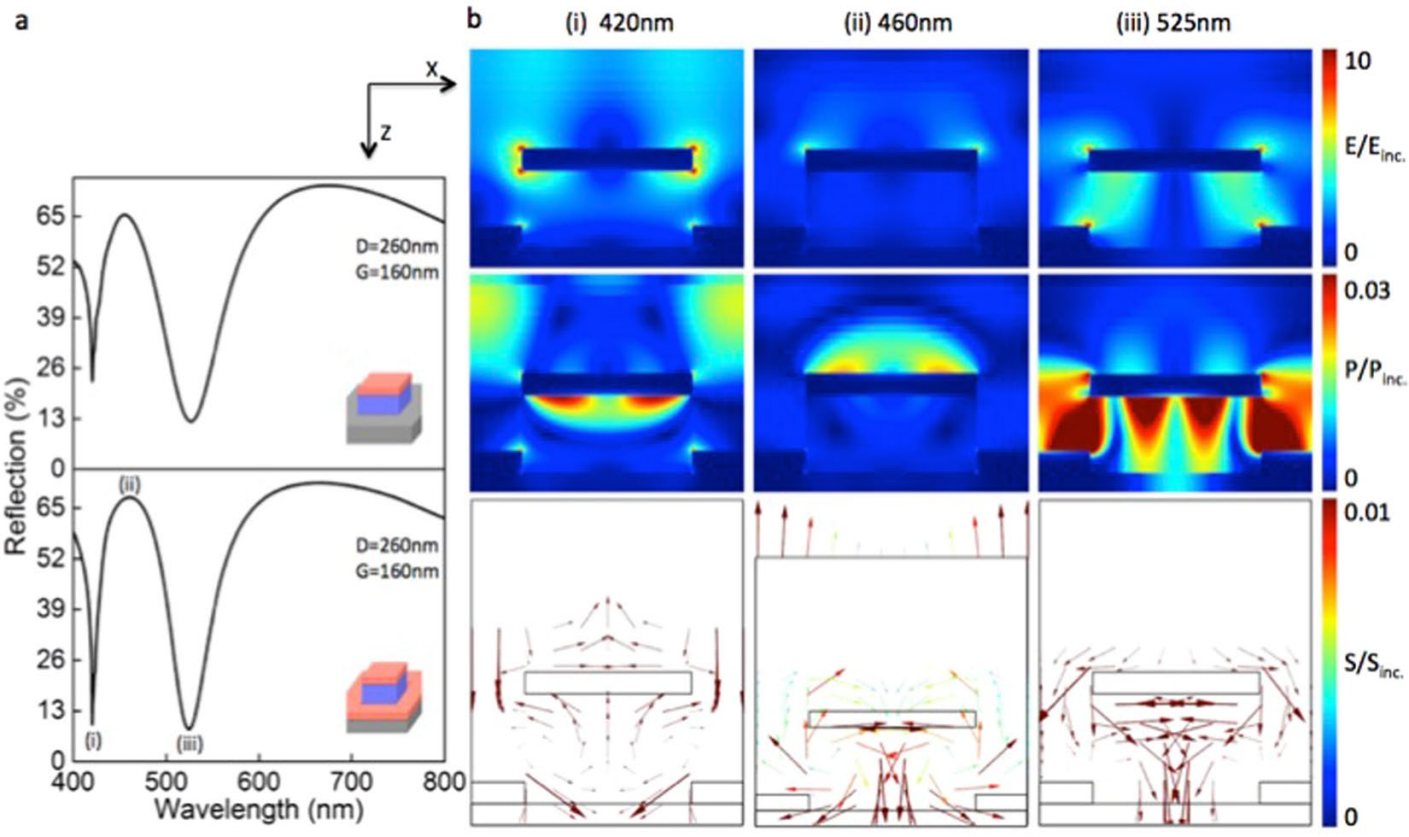
Numerical analyses of the spectral and electromagnetic behavior of the plasmonic structures using FDTD algorithm. (**a**) Calculated reflectance for the structures with (bottom) and without (top) back reflector, confirms the presence of multiple plasmonic resonances in both the cases. It is clear from the bottom figure that the strength of plasmonic light absorption increases with the presence of back reflector for both the resonances at (i) and (iii). (**b**) Electric field enhancement plots (top), time averaged power flow plots (middle) and Poynting vector plots (bottom) for the structure D = 260 nm with G = 160 nm at spectral positions marked in (**a**). (i) Dip 1 corresponds to the plasmonic resonance at 420 nm and it enhances the light absorption in to the substrate through the polymer nano pillar. (ii) Enhanced electric field due to the strongly radiating dipole resonance at 460 nm scatter coherently to form the strong reflective peak R_peak_. (iii) The Dip 2 corresponds to a strong LSPR at 525 nm enhances the electric field around the nano hole which in turn facilitates increased light absorption in to the substrate. In all simulations normal incidence of plane wave was carried out with the electric field polarized in the x-axis and the k-vector in the z-axis pointing from above.

As evident from the Fig. 5a the reflectance calculated for the structures (D = 260 nm and G = 160 nm) with and without back reflector exhibits light absorption at two distinct wavelengths for both the configurations, while the presence of a back reflector apparently increases the light extinction efficiency at the two resonance dips and scattering efficiency at the reflection peak (see Supplementary Fig. S9 for more details). The metal square patches in the 2D array structure play different roles in the appearance of different features in the reflection spectrum, which is explored in the Fig. 5b in which the electric field enhancement plots (top), time averaged power flow plots (middle) and Poynting vector plots (bottom) for the wavelengths 420 nm, 460 nm and 525 nm marked as (i), (ii) and (iii) in the bottom Fig. 5a are shown. The LSPR (LSPR) supported by the metal square patch facilitates the light absorption present at two distinct wavelengths marked as dips (i) and (iii). As shown in Fig. 5b, the electric filed enhancement at these wavelengths increases the light absorption by the polymer-substrate system. The Poynting vector plot clearly depicts the direction of the power flow in to the substrate through the nano hole^27^. The same metal square patch acts as a highly radiating dipole antenna for the peak reflection wavelength of 460 nm as shown in Fig. 5b(ii) ^8^. Corresponding Poynting vector plot shows the direction of the power flow in the positive z direction i.e. towards the observer. It can also be seen from the field and power enhancement plots that the back reflector also facilitates surface plasmon resonances for both the dips (i) and (iii), except for the reflection peak at (ii). This is the reason for the increased light absorption at the two dips in the combined structure shown in Fig. 5a.

**Figure 6:**
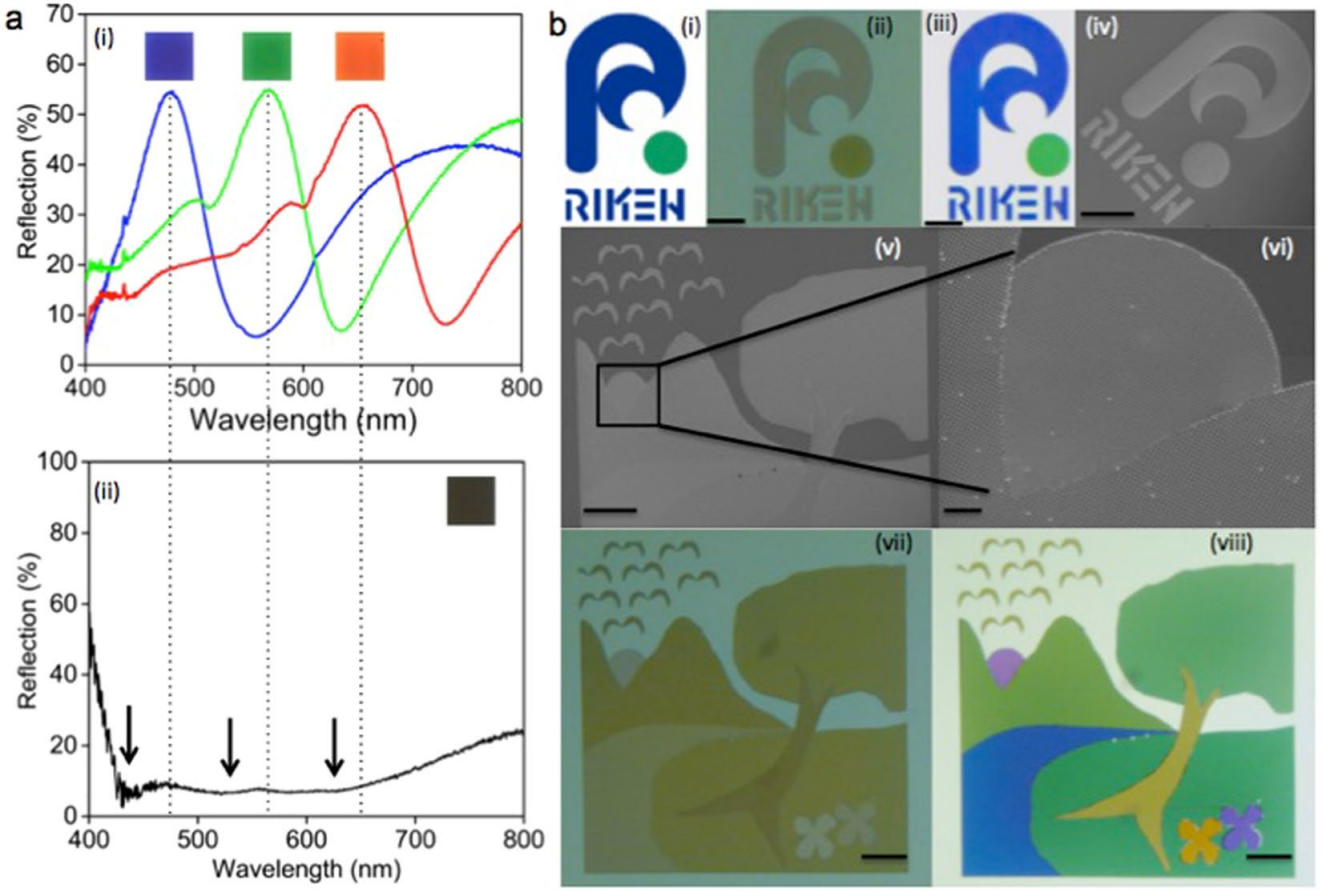
Full colour image printing and spectra of prominent colours red, green, blue and black. (**a**) Experimental reflection spectra for the (i) blue, green, red and (ii) black colour pallets. The black colour pallet is optimized using a broadband plasmonic light absorber consisting of metal structures of different sizes and the arrows in (ii) indicate the positions of resonant dips of individual plasmonic elements. The positions of peak reflections suppressed in (ii) is represented by the dotted trace lines. (**b**) Sub millimeter scale RIKEN logo and an artwork are printed using our colour mapping scheme to demonstrate the up scalable full colour image printing with high colour saturation. (i) Original RIKEN logo, (ii) lithographically patterned RIKEN logo in 300 μm square area before Al deposition, (iii) RIKEN logo after Al deposition and (iv) its SEM image. (v) SEM image of the art work printed in 300 μm square area using EBL, (vi) enlarged SEM image of the sun area, (vii) optical micrograph before Al deposition and (viii) that after Al deposition. All colour images are captured using a bright field optical microscope with 10x objective of NA 0.25. Scale bars: 50 μm (ii)–(v), (vii) and (viii) and 5 μm (vi).

A black colour is also highly desirable for any kind of full colour printing applications. The perception of a black colour is the result of the absence of light or zero light intensity in the reflected or transmitted plane. This could be achieved by designing a broadband absorber in the visible wavelength region. Near unity light absorption over a broad spectral range covering the entire visible region has already been demonstrated by combining multiple plasmonic resonant structures in a single pixel arrangement^28, 29^. Therefore, the requirement is that each constituent structure in the super pixel must possess non-overlapping resonances at distinct wavelengths to ensure broadband absorption^24^. We have designed a black colour pixel with constituent plasmonic structures having resonant dips at blue, green and red wavelengths, thus achieved near unity broadband absorption over the entire visible spectrum. The square shaped super pixel consists of two 180 nm, one 260 nm and one 340 nm square patches equally spaced by 150 nm in a 2D array structure (see Supplementary Fig. S10 for more details). The experimental reflection spectrum of the black colour pixel is shown in the Fig. 6a(ii) and the optical micrograph is shown in the inset. A reflection intensity of less than 10% is achieved for the entire visible wavelength region, which is also flat for a range of wavelength from 425–700 nm. The arrows in the Fig. 6a(ii) indicate the positions of resonance dips of the individual plasmonic elements, which are thus superposed to create the broad absorption spectrum rendering the black colour.

Now we demonstrate the printing of full colour images with sharp colour changes and tonal variations using this approach. Figure 6b(iii) and (viii) depicts the bright field optical micro images of the RIKEN logo and an art work printed in 300 μm square area using the colour pallets displayed in Fig. 2b. The high colour contrast achieved in these images underlines the advantages of using the proposed plasmonic geometry for full colour image printing. It can also be noted that only a single colour element is presented at the edge of the bird wings the smallest feature in the printed artwork, which is clearly distinguishable in the optical micrograph and indicative of the ability of a single nano structure to reflect individual colours (see Supplementary Fig. S11 for more details). A checkerboard consisting of alternating micro scale arrays of different colour pixels arranged in a square lattice was used for studying the closest colour resolution capability of our colour design strategy and the details are given in the Supplementary Information S12. The percentage reflection spectrum of the RGB colour pallets demonstrate narrow reflective peaks of high peak-valley ratio and intensity more than 50% as shown in the Fig. 6a(i), ideal for high contrast colour image printing. The sizes of the individual colour elements of blue, green and red colour pallets are 430 nm (D = 280 + G = 150 nm), 490 nm (D = 340 nm + G = 150 nm) and 600 nm (D = 420 nm + G = 180 nm) with corresponding peak reflection wavelength at 478 nm, 567 nm, and 657 nm, respectively. Relatively larger size of the individual colour elements^12, 18^, which is comparable to the wavelength of the respective reflected colours increases the fabrication tolerance and could be readily adopted in many commercial applications utilizing high throughput fabrication techniques employing NIL^30–32^ or laser interference lithography (LIL)^33, 34^.

## Conclusion

In conclusion, we have demonstrated a simple plasmonic geometry for ink free full colour printing with controllable colour attributes like hue, brightness and saturation. The colours are tunable across the visible spectrum by manipulating plasmonic light absorption at two separate wavelengths encoded in to the geometrical parameters of a single pixel. The colour attributes are preserved across the operating wavelength range by tuning the two resonances simultaneously. Independently tuning these resonances thus enables controlling the colour attributes at will. A black colour pixel with a flat reflection spectrum of intensity less than 10% is also demonstrated. The relatively larger size of individual colour elements consisting single metal structure of sub-micron size, considerably relaxes the fabrication requirements. Therefore, it is anticipated that our colour printing approach could be readily adopted in many practical applications utilizing high throughput NIL or LIL.

## Methods

### Electron beam lithography and pattern transfer

The nano structures comprising the plasmonic geometry, which displays vivid colours, were realized by electron beam lithography using Elionix ELS 7000 TR EBL system. The positive tone electron beam resist PMMA 950 A4 from Microchem was spin coated onto a piece of dry cleaned (100) silicon wafer, giving a thickness of around 150 nm. The resist was then prebaked at 180 °C for 30 sec. The use of positive tone PMMA resist reduced the effective exposure area considerably by exposing the narrow strip area which defines the inter particle spacing (gap G in the main text) leaving the wide area unexposed which defines the nano pillars after all processes. The computer generated layout consisting of horizontally and vertically arranged rectangular strips with equal spacing was prepared using Autocad software. The resist was then exposed with an acceleration voltage of 125 KV and a beam current of 100 pA was used. The write field was set to 300 μm × 300 μm with an exposure step size of 5 nm. These settings were used during optimization and the step size was increased to 15 nm for printing the images faster without compromising the print resolution and image quality, as the size of a single colour element was greater than 400 nm. A dose of 500 μC/cm^2^ was used for the exposure. The patterns were then transferred to the resist layer by developing it using a mixture of MIBK and IPA at 1:3 ratios at room temperature for 60 sec. followed by DI water rinsing for 30 sec. The samples were then blow-dried using a stream of N_2_.

### Metal deposition

High purity (5N) aluminum wire from Sumitomo Chemical Corp. was used as a source material for depositing the functional layer by thermal evaporation technique using a standard thermal evaporator TP-1 KC051 from Katagiri Eng. Corp. The chamber pressure during evaporation was maintained at ~1 × 10^−5^ Pa and a high coating rate of ~1 nm/sec. was used to reduce the roughness and hence to improve the surface morphology of the deposited thin film. The evaporation was done at room temperature without any stage rotation and tilt angle normal to the substrate surface.

### Optical measurements and surface profiling

The spectral and colour information of each plasmonic colour pixel were captured by micro-spectroscopic technique using Olympus BX51 series optical microscope coupled with a USB2000 + XR1-ES optical spectrometer having an operating wavelength range of 200–1025 nm from Ocean Optics and a 3MP CCD camera, MOTICAM2300 from Shimadzu Corp. respectively. Unpolarized white light from a halogen lamp was normally incident onto the pixels using a standard objective lens of magnification 20x and numerical aperture 0.4. The reflected light was collected by the same objective and one portion was directed to the CCD camera for extracting the colour information and the other portion to the spectrometer for extracting the spectral information. The reflection spectra of each colour pallet were normalized with that of a reference sample with an aluminum mirror of 200 nm thickness deposited on a silicon substrate. The optical micrographs were captured using the Olympus digital video camera Polaroid PDMC11/OL with an Olympus BX51 M setup.

The morphology and dimensional parameters were analysed using a scanning electron microscope S-4800T from Hitachi with an acceleration voltage of 5 kV and a working distance of 8 mm. The height of the PMMA pillars was accurately measured using the Bruker’s Dimension Icon atomic force microscope (AFM). This height information is crucial for the numerical modeling of the nano structures.

### Numerical modeling

A finite difference time domain (FDTD) model of the nano structure was analyzed using Lumerical’s FDTD solutions. The frequency dependent permittivity of silicon and aluminum was taken from the reference^35^, which is also available in the material library of the software. The PMMA nano pillars were modeled using a refractive index of 1.49. Periodic boundary conditions were implemented in the x and y directions, which are normal to the sample surface and perfectly, matched layer (PML) boundary conditions were implemented in the xy plane on top and bottom of the sample surface. The reflectance was calculated using normal incidence of plane waves onto the sample. The height of the PMMA nano pillars was set to 150 nm as obtained from the AFM measurement.

## Electronic supplementary material

Supplementary Information
