# Supplementary material for: Up Scalable Full Colour Plasmonic Pixels with Controllable Hue, Brightness and Saturation
Source: Sci Rep. 2017 Apr 26;7:1199. doi: 10.1038/s41598-017-01266-6 (PMC5430921; doi:10.1038/s41598-017-01266-6)
Supplement: Supplementary file 1 — Supplementary Information [file 41598_2017_1266_MOESM1_ESM.pdf]

Up Scalable Full Colour Plasmonic Pixels with Controllable Hue, Brightness and Saturation

Renilkumar Mudachathi[1] and Takuo Tanaka[1,2,3*]

Supplementary information

S1

The simulated reflection spectra of the plasmonic structures in the 2D array structure for varying pillar height *h* (the inset shows the predicted colours from the respective spectrum). It can be seen from the left figure that the light extinction efficiency at these two resonance positions reaches to a maximum value for an optimum pillar height. The higher order resonance was started to appear at a pillar height of 100-nm and the near unity light absorption happens at a pillar height of 200-nm. After that the strength of the absorption was started to decrease. The figure shown at the right depicts the traits of the resonance wavelength shift with respect to the changing *h*.

It is clear from the reflection spectra that the line width of the reflective peak around 450-nm is narrower for a pillar height between 140 to 160-nm, beyond the pillar height of 160-nm, the reflection peak broadens considerably. This line broadening will lead to poor colour saturation. The increased colour saturation observed in the predicted colour (shown in the inset of Fig. S1(a)) at a pillar height of 200-nm may be attributed to the

increased light absorption for the two resonance peaks at 420-nm and 525-nm.

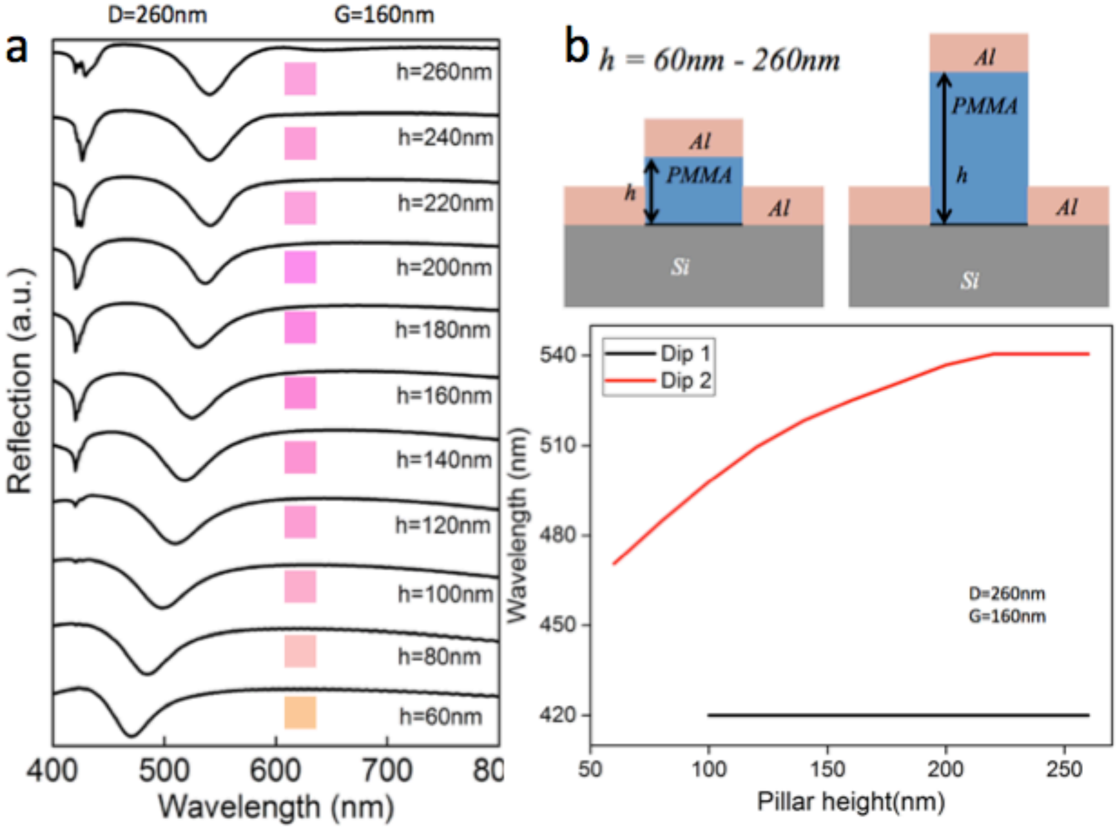

S2

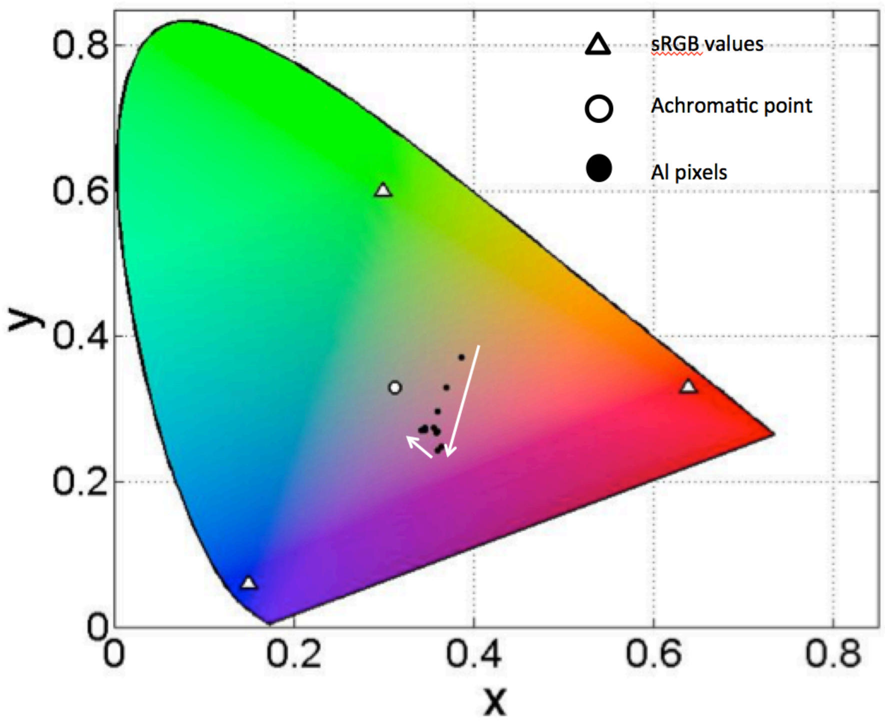

The colour values extracted from the spectra for different pillar heights are plotted in the CIE1931 colour gamut and shown in the Fig. S2. The white arrows indicate the trend of the colour evolution. For the pillar height from 60-nm to 200-nm the colour saturation increases (white arrow pointing downwards) and for the pillar height above 200-nm the colour saturation decreases (white arrow pointing upwards). The white triangles represent the sRGB values and the white circle represents the achromatic point.

S3

AFM measurement of the pillar height and in the inset shows the AFM image of the sample. The pillar height was measured to be 150-nm and all calculations are carried out using this pillar height.

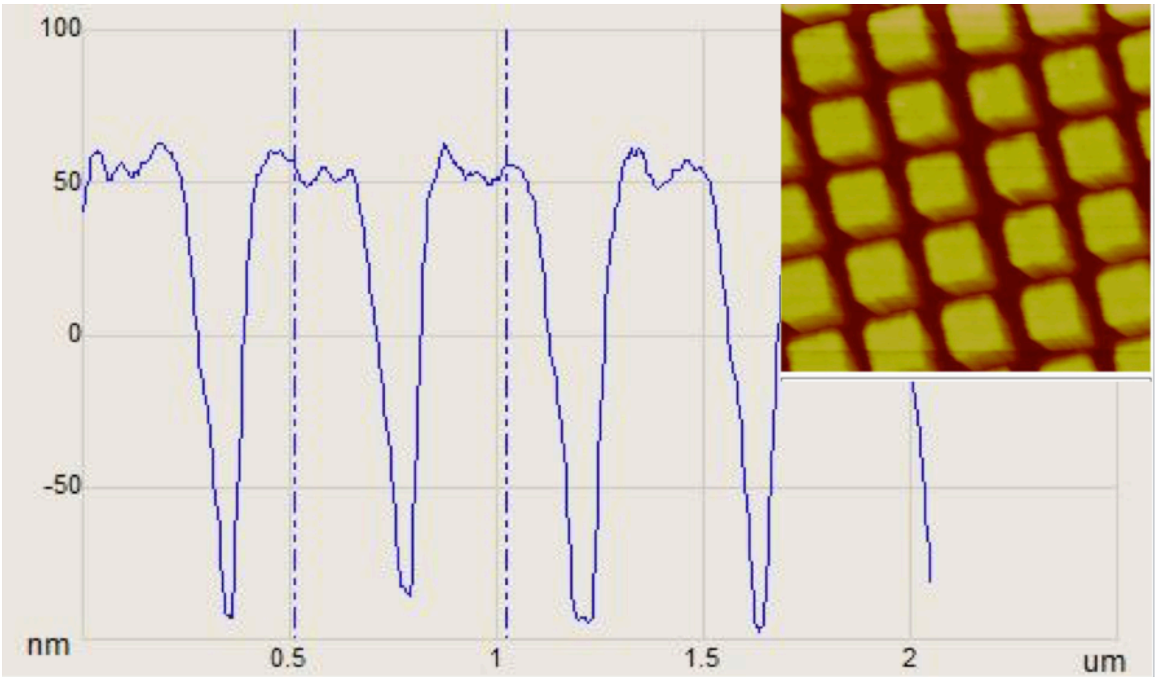

S4

Experimental reflectance of all colour pallets displayed in Figure 2b of the main text. The vertical axis of each figure is the gap size G, which varies from 100-180-nm with an increment of 10-nm. Respective colours are also shown in the inset.

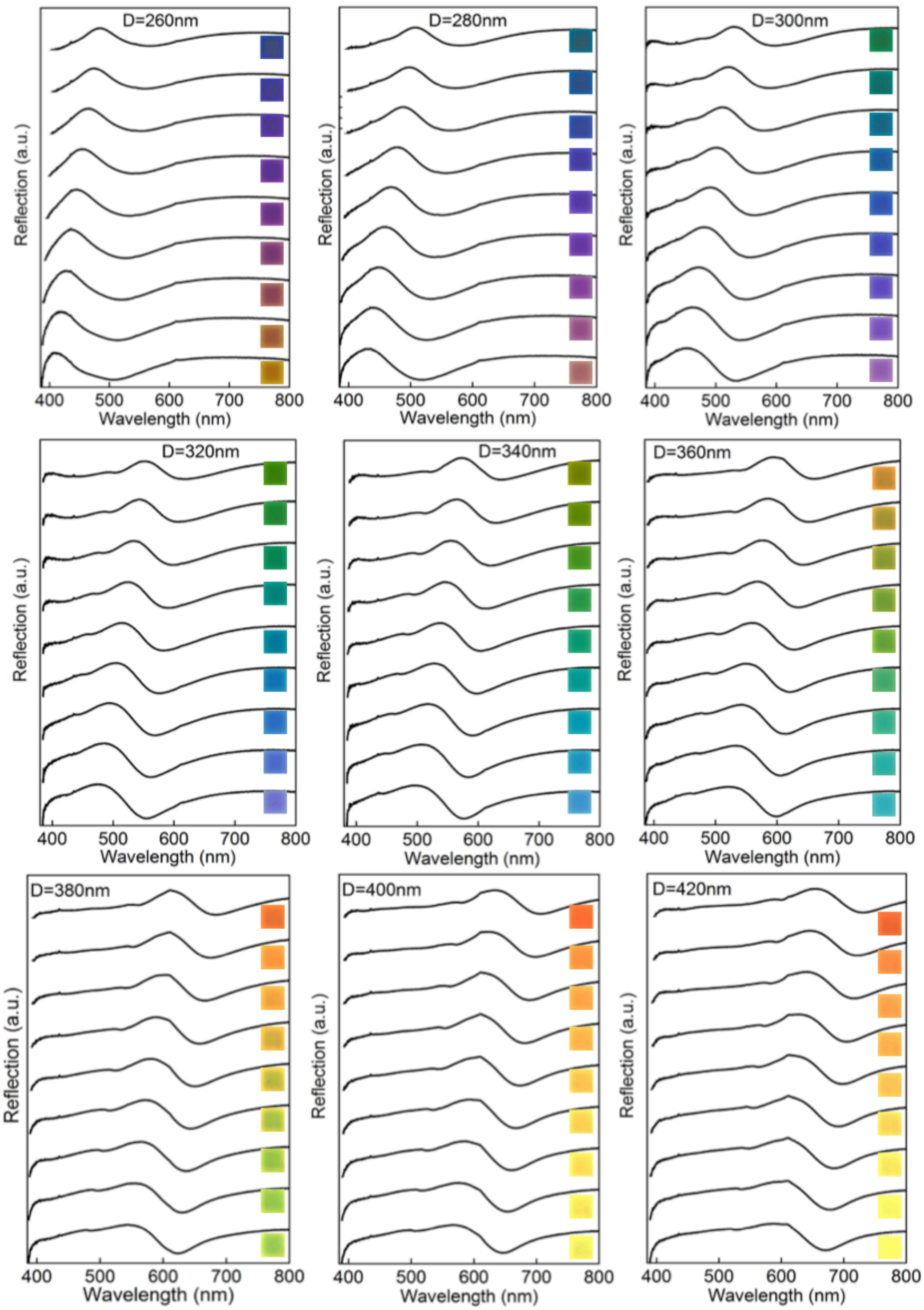
D=260nm
D=280nm
D=300nm
D=320nm
D=340nm
D=360nm
D=380nm
D=400nm
D=420nm
Reflection (a.u.)
Wavelength (nm)
400
500
600
700
800

S5

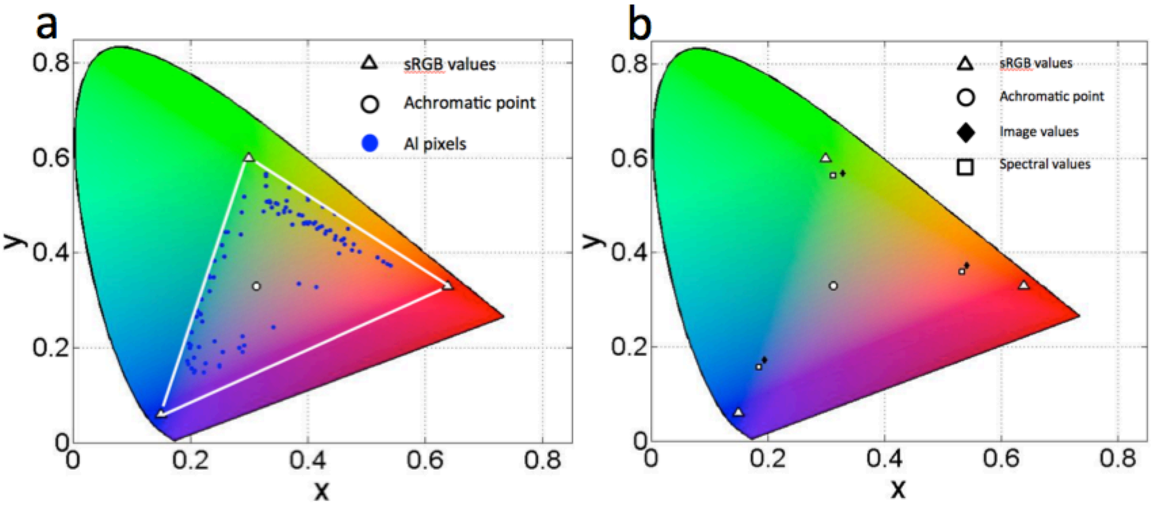

c

	R	G	B
x	0.5421	0.329	0.1951
y	0.3738	0.5679	0.1722
Y	32.139	29.857	19.318

d

	R	G	B
x	0.533	0.313	0.1854
y	0.36	0.5631	0.1567
Y	33.283	31.53	18.009

The tristimulus values have been extracted from the experimental spectra for red, green and blue pixels of dimension D/G = 420-nm/180-nm, 340-nm/180-nm and 260-nm/180-nm respectively. We found negligible deviation between the values obtained from the spectral information and the optical images of the corresponding colour pixels. The tristimulus values obtained by the two methods are plotted in the CIE1931 colour gamut and shown in the Fig. S5 (b), in which both the values are plotted together with sRGB values for better comparison. The sRGB colour gamut is shown in the Fig. S5 (a) (area enclosed in the white lines) along with the x-y positions of the aluminum colour pixels (blue circles), which are well aligned with the white lines far from the achromatic point (white circle) confirms the capability of the present colour design strategy to produce all colours in the

visible spectrum with high colour purity. The x and y values with corresponding colours extracted from the optical images (c) and that from the spectra (d) for red, green and blue pixels are also highlighted in the Fig. S5 for comparison between two methods used for colour value calculation.

S6

The dependence of incident angle on the reflection spectra has been studied using FDTD method. The model used in this simulation has dimensions D=260-nm and G=170-nm giving a periodicity of 430-nm. The results are shown below,

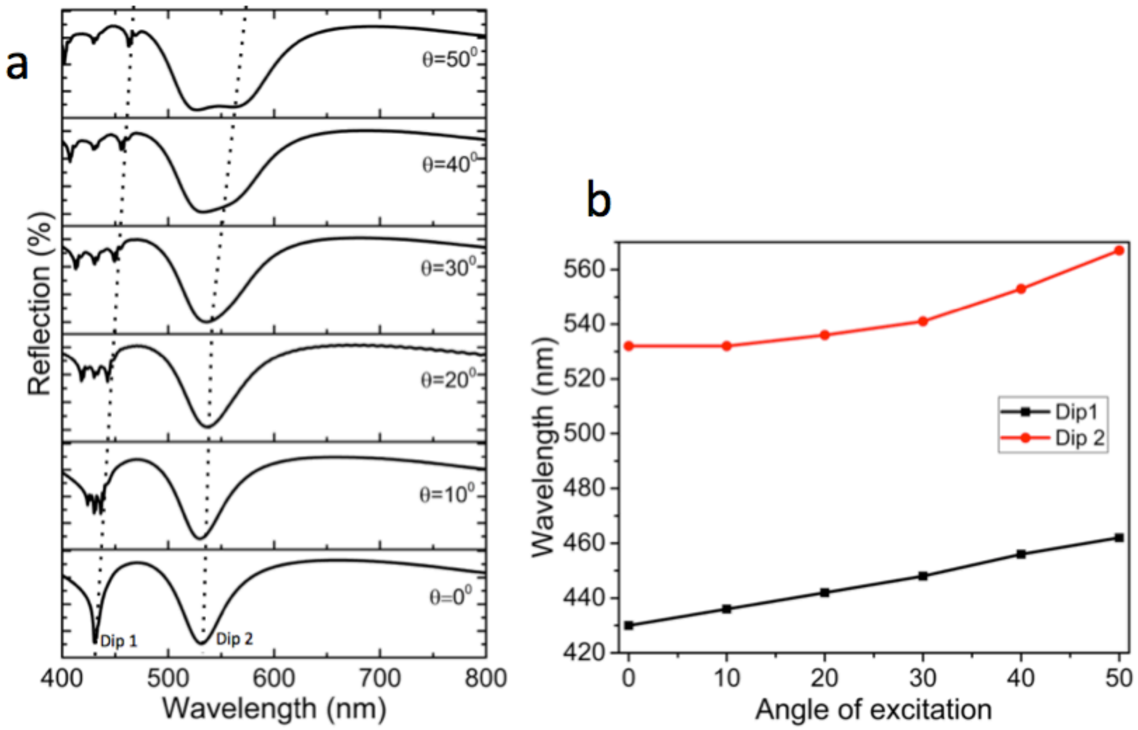

It can be seen from the above plot (a) that additional peaks are emerging for an angle above 10º at wavelengths 400-500-nm range, which can be attributed to the grating-assisted excitation of surface plasmons at the aluminum PMMA interfaces. It is also worth noting that for an angle above 30º, the resonance dip at 520-nm is started to broaden and split in to two dips (seen only above an angle of 65º). These resonance dips can only be observable under oblique incidence and may be captured in the measured spectra as the high N.A. objective used in the measurement can support many angles of excitation. The LSPR at around 530-nm is invariant up to an angle of 30º (less than 0.2-nm/degree at the resonance peak). And after that it started to broaden and show clear angular dispersion (b). The wavelength shift of the resonance at 430-nm is almost linear (around 0.9/degree).

S7

Experimental (a) and simulated (b) reflection spectra for the structures with same pixel size of 480-nm. The respective colours shown in the inset confirms the controllable colour saturation with changing gap size by keeping the pixel size same.

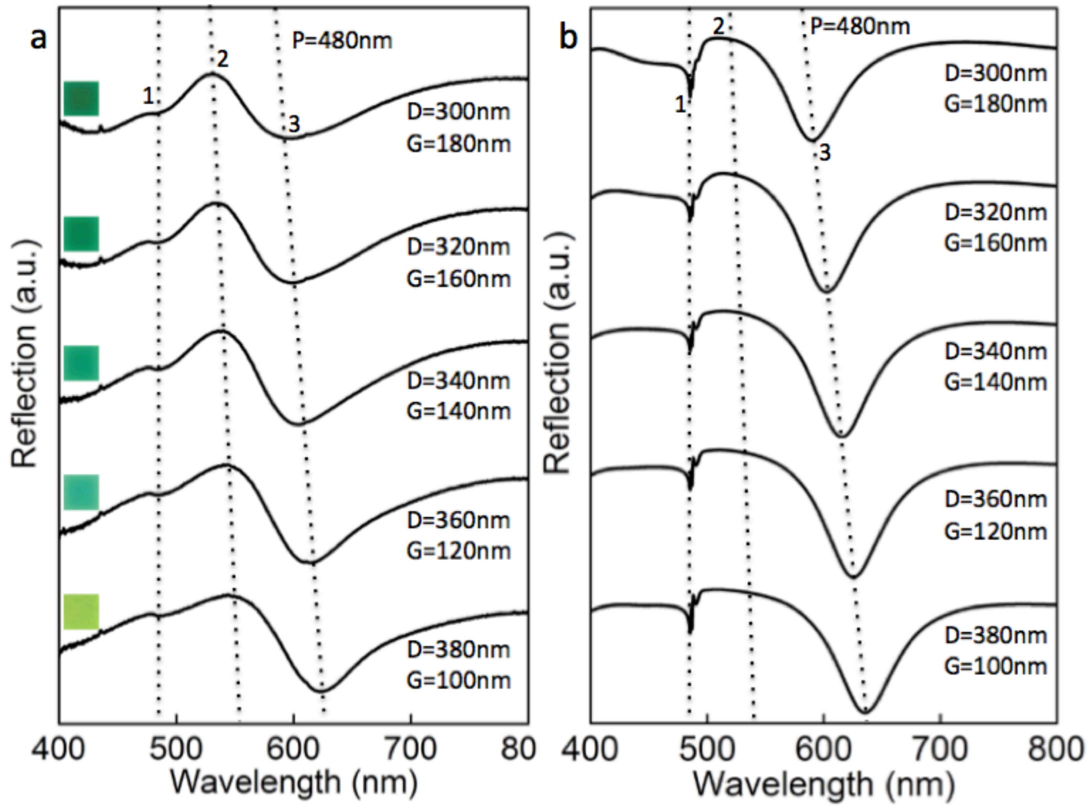

S8

Experimental (a) and simulated (b) reflectance of the structures with same P, showing increasing peak reflection intensity with decreasing G, indicative of controllable colour brightness.

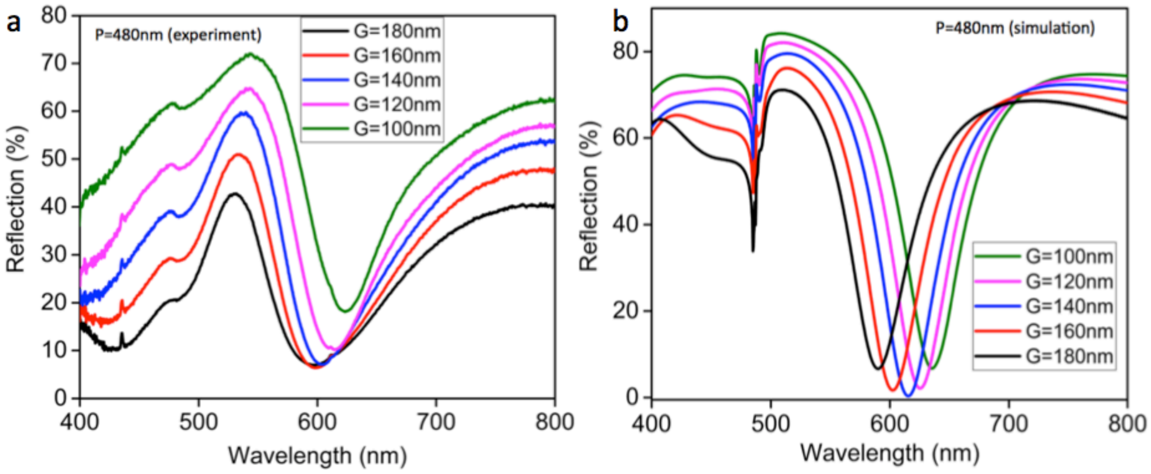

S9

Simulated reflectance of the structures with (black line) and without (red line) back reflector as a function of D (left) and G (right). The plasmonic light absorption is increased with the presence of back reflector indicative of surface plasmon resonance supported by the holey Al back reflector.

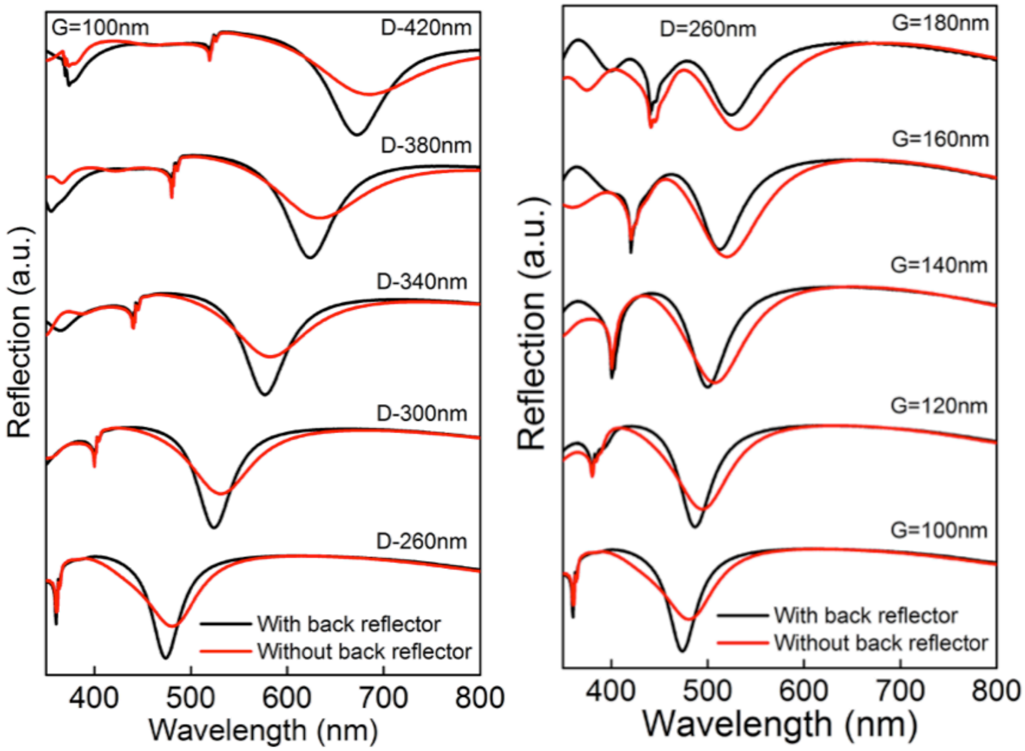

S10

The black colour super pixel is constructed using metal square patches of different sizes arranged together in a single unit cell. Each metal patch is

designed to possess plasmonic resonances at individual wavelengths spanning the entire visible wavelength region. The square shaped super pixel consists of two 180-nm, one 260-nm and one 340-nm square patches equally spaced by 150-nm in a 2D array structure. The SEM image of the black colour pixel is shown below in the Fig. S10 (b). In the unit cell, apart from two 180-nm, one 260-nm and one 340-nm square patches a 100-nm square patch is also included to reduce the unoccupied area of the back reflector so that the unwanted reflection could be avoided. The 180-nm, 260-nm and 340-nm square patches are expected to have resonance dips in the blue, green and red region of the visible spectrum so that a flat reflection valley could be achieved.

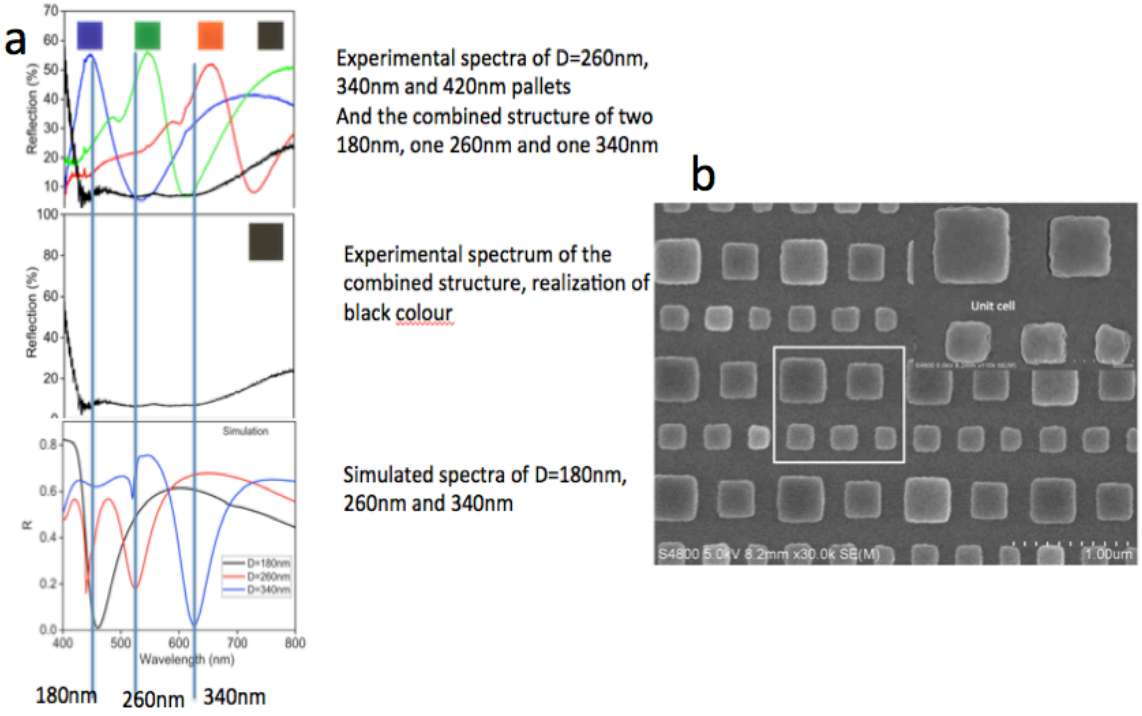

Experimental spectra of RGB and black colour pixels along with the

calculated reflectance of the individual metal structures in the black colour

super pixel are shown in (a). The blue lines trace the peak and dip positions in the experimental and calculated spectra indicate the absorption of blue, green and red colours together to produce the black ciolour pixel.

The 180-nm metal structure has a resonance at 460-nm, the 260-nm structure has resonance at 520-nm and the structure 420 has the resonance at 625-nm. Which cuts off blue, green and red colours from the reflection spectrum.

S11

Optical micrograph of the enlarged image of the smallest feature. It can be noted that only a single colour element is presented at the edge of the bird wings the smallest feature in the printed art work, which is clearly distinguishable in the optical micrograph and indicative of the ability of a single nano structure to reflect individual colours.

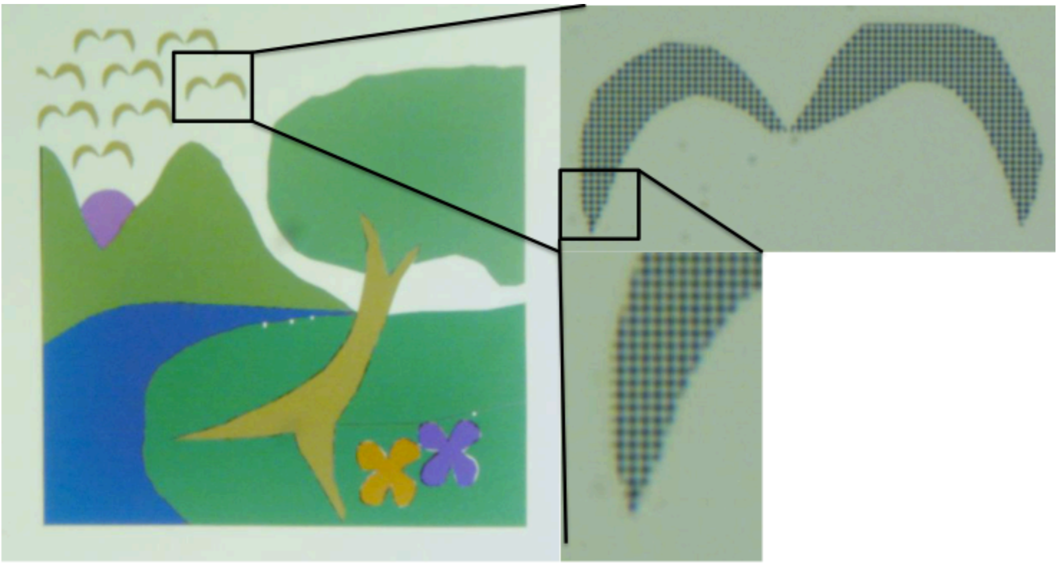

S12

A closest colour resolution study has been performed by designing a checkerboard with different colour pixels arranged in a square lattice as shown below,

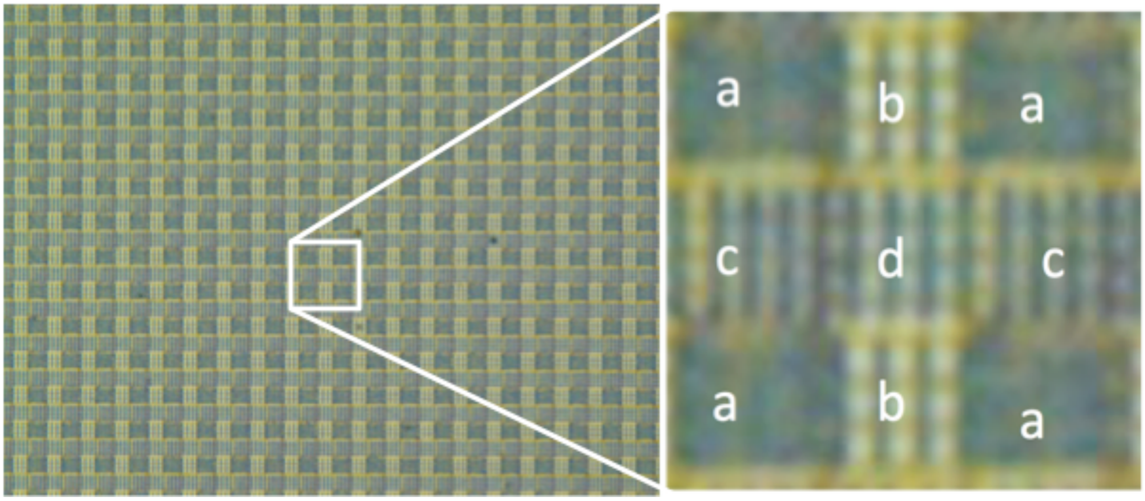

Metal structures of D/G = 260-nm/180-nm (a), 320-nm/180-nm (c), 360-nm/180-nm (d) and 420-nm/180-nm (b) are used for the checkerboard design. 2μm × 2μm arrays of different colour pixels are alternately arranged in a 2D square lattice of size 200μm × 200μm as shown in the above high-resolution optical image.

The figures (a) and (b) shown below depicts the high resolution (100× objective) and low-resolution (20× objective) optical images of a small portion of the 200μm × 200μm array respectively. It is to be noted that at high resolution the individual structures are resolved well but at lower resolution (20x) the 2μm × 2μm pixel displays the individual colour. The scale bars are different for the images shown in the figures (a) and (b). The image shown in the figure (b) is an enlarged image to fit with that shown in (a). The actual size of the low resolution image is shown in the figure (c) for comparison.

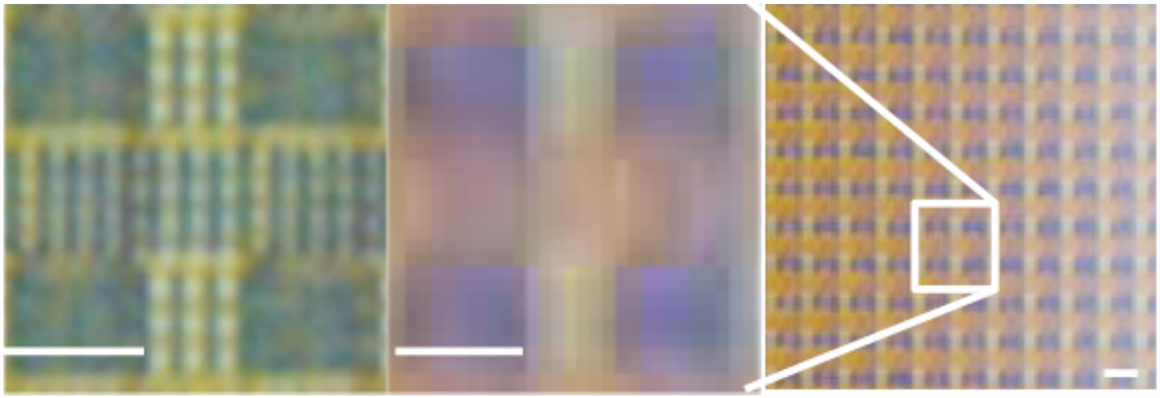

(a) (b) (c)

(a) High resolution optical image of a small portion of the checkerboard, (b) enlarged low resolution optical image of the same area of the checker board and (c) a size comparison of the two images (scale bar in all figures is 2μm).
